# INTIMATE PARTNER HOMICIDE SUICIDE IPHS EVENTS AND MEDIA COVERAGE: PANDEMIC COMPARED TO PREPANDEMIC YEARS

**DOI:** 10.1093/geroni/igae098.2333

**Published:** 2024-12-31

**Authors:** Sonia Salari, Emma Videla, Carrie Sillito

**Affiliations:** University of Utah, Salt Lake City, Utah, United States; University of Utah, Salt Lake City, Utah, United States; University of Utah, Salt Lake City, Utah, United States

## Abstract

Intimate partner homicide-suicide IPHS, involves the murder of a current or former partner, followed by the offender’s suicide. We content analyzed our news surveillance data over the modern pre-pandemic years (2018, 2019) and pandemic years (2020-2022). In 2020, U.S. domestic violence went up 8% and the FBI reported a ‘murder surge.’ Lockdown reduced COVID contagion, but also isolated people in their homes, some with abusers. FBI NICs data on background checks show a pandemic run on firearm acquisitions in U.S. households. We collected articles, TV news, police reports, and obituaries for n=725 IPHS events, representing over 1600 deaths. Age categories are young (18-44, n=256), middle (45 to 59, n=176) and elder adult cases, age 60+ (n=293). Evidence suggests gender-based violence in the form of femicide, as the vast majority (95%) were male perpetrated. Our research questions: 1) Did 2020 have more IPHS? and 2) What qualitative changes in IPHS media coverage happened during the pandemic? Our results found a dip in IPHS for the year 2020, but increasing violence in 2021 and 2022 (peak)—particularly for the young. We suggest a “crisis effect” for IPHS in 2020 and then a surge. Pandemic gun sales were greater among the young. Media coverage of IPHS varied by age. Elder adults had significantly fewer cases with names announced in news media, especially during the pandemic. This represents ageism, because the young and middle-aged had almost none who went unannounced. Domestic violence victims of all ages deserve recognition.

